# Impacts of Replacing Fish Meal With Duck By‐Product Meal in Diets on Growth Performance, Feed Utilization, and Economic Efficiency of Olive Flounder (*Paralichthys olivaceus*)

**DOI:** 10.1155/anu/3065283

**Published:** 2026-06-18

**Authors:** Seong Woo Shin, Sung Hwoan Cho

**Affiliations:** ^1^ Ocean Science and Technology School, Korea Maritime and Ocean University, Busan, 49112, Republic of Korea, kmou.ac.kr; ^2^ Division of Convergence on Marine Science, Korea Maritime and Ocean University, Busan, 49112, Republic of Korea, kmou.ac.kr

**Keywords:** duck by-product meal, economic efficiency, fish meal substitution, low-fish meal diets, *Paralichthys olivaceus*

## Abstract

Fish feeds are generally more expensive than livestock feeds because they contain high amounts of costly fish meal (FM). Therefore, formulating low‐FM diets is necessary for the sustainability of aquaculture. This study evaluates impacts of FM replacement with duck by‐product meal (DBM) in diets for olive flounder (*Paralichthys olivaceus*) on growth performance, feed utilization, and economic efficiency. Four hundred and fifty juvenile fish (initial weight of 6.30 g) were equally distributed into 18, 50‐L flow‐through tanks in triplicate. Six experimental diets were formulated to be isoproteic (52.0%) and isolipidic (12.0%). The control (Con) diet contained 60% FM. In the Con diet, 20%, 40%, 60%, 80%, and 100% of FM were substituted with DBM, named as the DBM20, DBM40, DBM60, DBM80, and DBM100 diets, respectively. Fish were hand‐fed to apparent satiation throughout the 50‐day trial. After the 50‐day feeding trial, weight gain (WG), specific growth rate, and feed consumption were significantly (*p* < 0.0001 for all) higher in fish supplied with the Con, DBM20, and DBM40 diets than in those supplied with the DBM60, DBM80, and DBM100 diets. However, there were no significant (*p* > 0.05) differences in feed utilization, plasma chemistry, and whole‐body fish supplied with the experimental diets. Amino acid and fatty acid profiles of the whole‐body fish were strongly influenced by dietary amino acid and fatty acid profiles. Economic profit index (EPI) of the Con, DBM20, and DBM40 diets was significantly (*p* < 0.0001) higher than that of the DBM60, DBM80, and DBM100 diets. Therefore, DBM could replace up to 40% of FM in a 60% FM‐based diet without negative impacts on the growth performance, feed utilization, plasma chemistry, and chemical composition of olive flounder and EPI.

## 1. Introduction

Seafood consumption has been widely promoted because of its well‐established health benefits, and global consumption of seafood has increased steadily over the past few decades [1]. In response to this growing demand, aquaculture production has expanded and become an increasingly important component of the global food systems [2]. According to FAO [3], aquaculture production in the Republic of Korea (henceforth, Korea) has increased from ~0.29 million metric tons (MT) in 1980–0.56 MT in 2023. Thus, the Korean government has actively promoted the aquaculture industry as a key strategy to satisfy domestic demand for seafood [4].

In Korea, olive flounder (*Paralichthys olivaceus*) recorded the highest aquaculture production (40,125 MT), accounting for 49% of total aquaculture production (81,911 MT) and value (684 billion KRW), accounting for 57% of total aquaculture value (1211 billion KRW) among marine finfish species in 2024 [5]. This indicates that olive flounder has become the most commercially important marine fish species for aquaculture in Korea.

In intensive aquaculture systems including olive flounder culture, fish rely largely on supplied feeds as the main source of nutrients and energy required for growth, reproduction, health maintenance, and other physiological functions [6]. Growth rate and feed conversion ratio (FCR) are the key determinants of profitability in fish farming, because faster growth increases overall production and improved FCR reduces feed‐related costs [7]. Animal tissue contains protein as its principal organic constituent [8]. Approximately 65%–75% of protein on a dry basis is a main component of the total body weight of fish, and formulated feeds should therefore contain ca. 31%–55% dietary protein to support optimal growth in fish [9]. However, the high protein content in fish feeds substantially increases feed costs, thereby elevating overall production costs in aquaculture [10]. Therefore, lowering the cost of protein sources in formulated feeds is critical for minimizing feed costs and enhancing the sustainability of aquaculture.

In general, carnivorous fish species including olive flounder are known to have relatively high dietary protein requirement for optimal growth compared to either omnivorous or herbivorous fish species [11]. Fish meal (FM) is a main protein source in formulated fish feeds because it is abundant in essential amino acids (EAAs) and fatty acids (EFAs), including n‐3 highly unsaturated FA (n‐3 HUFA), and has high digestibility and palatability [12, 13]. However, the global production of FM has been plateaued [14] due to overfishing of FM resources and the occurrence of abnormal climate changes [15]. Meanwhile, with the continued expansion of the aquaculture industry, the demand for FM has increased [16], leading to a continuous rise in international FM price [17]. Therefore, active research has been globally performed on the potential use of dietary FM replacements with animal by‐product meals, such as meat meal [18], meat and bone meal [19], poultry by‐product meal (PBM) [20], blood meal [21], and fish processing by‐product meal [22], which are less expensive than FM and available year‐round. In addition, substitutability of insect meal for FM in olive flounder has been also studied [23].

Duck by‐product meal (DBM), a type of PBM, is produced by drying and grinding the solid residues obtained after oil extraction from duck processing by‐products including heads, feet, internal organs, blood, bones, and feathers, accounting for ~28%–30% of total body weight per duck [24]. It contains high crude protein (over 60%) and crude lipid (over 10%) levels, and a higher arginine content (3.9% in the diet) compared to FM (3.5% in the diet) [25]. In addition, it is available at a much lower price (USD 0.54/kg; USD 1 = 1350 KRW) than FM (USD 2.37/kg) [25]. From 1995 to 2023, global annual duck meat production increased steadily from 2.1 to 7.1 million MT, accompanied by a rise in the number of slaughtered ducks from 1.4 to 4.2 billion heads, suggesting a stable and expanding long‐term supply [26]. In particular, global duck meat production reached 6.2 million MT in 2023, with Korea producing 74,968 MT, placing the country within the top 10% of duck‐producing countries worldwide [26]. Accordingly, DBM can be considered a potential protein ingredient as a FM replacer in olive flounder diets.

Numerous studies have evaluated PBM as a FM replacer in fish feeds. In olive flounder, up to 50% of FM could be substituted with chicken by‐product meal (CBM) in a 65% FM‐based diet [27]. In Asian seabass (*Lates calcarifer*), up to 75% of FM was reported to be replaceable by either PBM or processed PBM in a 61% FM‐based diet [28]. Collectively, these studies suggest that various PBM could serve as a promising alternative protein source for FM in fish feeds.

However, to date, no studies have evaluated the substitutability of DBM as a sole alternative protein source for FM in aquafeeds. The present study, thus, aims to evaluate the replacement impacts of graded levels FM with DBM in diets on the growth performance and feed utilization of olive flounder, and economic analysis.

## 2. Materials and Methods

### 2.1. Rearing Conditions

Juvenile olive flounder were bought from a private hatchery (Hyun Susan; Buan‐gun, Jeollabuk‐do, Korea) and acclimatized to a 3‐ton square flow‐through tank. Before starting the feeding experiment, fish were provided with the commercial diet containing 52% crude protein and 10% crude lipid (Jeil Feed Co., Ltd., Haman‐gun, Gyeongsangnam‐do, Korea) twice a day for 2 weeks. After the 2‐week acclimation period, total of 450 juvenile fish (6.30 ± 0.072 g; mean ± SE) were equally distributed into 18, 50‐L flow‐through tanks (water volume: 40 L) (25 fish per tank).

Each tank was supplied with drum‐filtered seawater at a flow rate of 2.8 L/min along with steady aeration. Water quality was monitored once a day using a multifunctional water quality meter (AZ‐8603; AZ Instrument Corp., Taichung, China). The temperature, dissolved oxygen, salinity, and pH ranged from 18.0°C to 24.4°C (20.8°C ± 1.47°C; mean ± SD), from 7.1 to 8.1 mg/L (7.6 ± 0.22 mg/L), from 33.0 to 36.7 g/L (34.8 ± 1.05 g/L), and from 8.0 to 8.4 (8.2 ± 0.10), respectively.

### 2.2. Preparation of the Experimental Feeds

The control (Con) diet included 60% FM and 16% fermented soybean meal as the main protein sources, 15% wheat flour as the main carbohydrate source, and 4% fish oil and 2.5% soybean oil as the lipid sources, respectively (Table 1). In the Con diet, 20%, 40%, 60%, 80%, and 100% of FM were replaced with DBM, named as the DBM20, DBM40, DBM60, DBM80, and DBM100 diets, respectively. All experimental diets were formulated to be isoproteic at 52.0% and isolipidic at 12.0%.

**Table 1:** Ingredient and chemical composition of the experimental diets (%, DM basis).

Ingredients (%, DM)	Experimental diets					
	Con	DBM20	DBM40	DBM60	DBM80	DBM100
Fish meal^a^	60.0	48.0	36.0	24.0	12.0	—
Duck by‐product meal^b^	—	14.0	28.0	42.0	56.0	70.0
Fermented soybean meal	16.0	16.0	16.0	16.0	16.0	16.0
Wheat flour	15.0	13.5	12.0	10.5	9.0	7.5
Fish oil	4.0	4.0	4.0	4.0	4.0	4.0
Soybean oil	2.5	2.0	1.5	1.0	0.5	—
Vitamin premix^c^	1.0	1.0	1.0	1.0	1.0	1.0
Mineral premix^d^	1.0	1.0	1.0	1.0	1.0	1.0
Choline	0.5	0.5	0.5	0.5	0.5	0.5
*Nutrients* (*%*, *DM*)						
Dry matter	95.7	95.0	95.1	96.6	96.4	95.9
Crude protein	52.3	52.5	52.5	52.0	52.4	52.3
Crude lipid	11.9	11.9	11.5	11.6	11.7	11.8
Ash	10.3	12.4	13.9	15.5	16.5	17.0
Gross energy (kcal/g)	4.6	4.6	4.6	4.6	4.6	4.5

^a^Fish meal (FM; crude protein: 69.8%, crude lipid: 7.8%, and ash: 14.6%) imported Chile (USD 2.37/kg FM, USD 1 = 1350 KRW).

^b^Duck by‐product meal (DBM; crude protein: 61.6%, crude lipid: 10.6%, and ash: 21.2%) was purchased from Daehan Feed Co., Ltd. (Incheon Metropolitan City, Korea) (USD 0.54/kg DBM).

^c^Vitamin premix (g/kg mix): L, ascorbic acid; 121.2; DL‐α‐tocopheryl acetate, 18.8; thiamin hydrochloride, 2.7; riboflavin, 9.1; pyridoxine hydrochloride, 1.8; niacin, 36.4; Ca‐D, pantothenate; 12.7; myo‐inositol, 181.8; D, biotin; 0.27; folic acid, 0.68; p‐aminobenzoic acid, 18.2; menadione, 1.8; retinyl acetate, 0.73; cholecalciferol, 0.003; cyanocobalamin, 0.003.

^d^Mineral premix (g/kg mix): MgSO_4_·7H_2_O, 80.0; NaH_2_PO_4_·2H_2_O, 370.0; KCl, 130.0; ferric citrate, 40.0; ZnSO_4_·7H_2_O, 20.0; Ca‐lactate, 356.5; CuCl, 0.2; AlCl_3_·6H_2_O, 0.15; KI, 0.15; Na_2_Se_2_O_3_, 0.01; MnSO_4_.H_2_O, 2.0; CoCl_2_·6H_2_O, 1.0.

All ingredients were mixed with water at a 3:1 ratio using a vertical mixer (B20GA; Eben Commerce Korea, Ansan‐si, Gyeonggi‐do, Korea). The mixture was shaped into pellets using the extruder (SMC‐32; SL Company, Incheon Metropolitan city, Korea) with diameters of 2–3 mm, considering mouth size of olive flounder. Subsequently, all experimental diets were dried using a machine (JW‐1350ED; Jinwoo Electronics Co., Ltd., Hwaseong‐si, Gyenggi‐do, Korea) at 50°C for 48 h and then stored at −20°C until feeding.

All experimental feeds were assigned to triplicate groups of fish. Experimental fish were carefully hand‐fed to apparent satiation twice a day (07:00 and 16:00) for 50 days. All tanks were siphon‐cleaned daily after morning feeding, and the photoperiod followed the natural light cycle. The daily amount of feed provided to each tank was recorded. Dead fish were promptly removed upon observation.

### 2.3. Measurements of the Biological Indices of Fish

After the 50‐day feeding experiment, all surviving fish were fasted for 24 h and then anesthetized with tricaine methanesulfonate (MS‐222) at a concentration of 100 mg/L. All surviving fish in each tank were counted and weighed collectively to calculate survival rate and weight gain (WG). From each tank, randomly selected 10 fish were anesthetized, and individually weighed and measured for total length. The viscera and liver were then collected to determine the viscerosomatic index (VSI) and hepatosomatic index (HSI). Growth performance, feed utilization, and biological indices of fish were calculated as follows: specific growth rate (SGR, %/day) = [Ln final weight of fish (g) − Ln initial weight of fish (g)] × 100/days of feeding (50 days), Feed efficiency (FE) = [total final weight of fish (g) − total initial weight of fish (g) + total weight of dead fish (g)]/total feed consumption (FC, g), protein efficiency ratio (PER) = WG of fish (g/fish)/protein consumption of fish (g/fish), protein retention (PR, %) = protein gain of fish (g/fish) × 100/protein consumption of fish (g/fish), condition factor (CF, g/cm^3^) = body weight of fish (g) × 100/total length of fish (cm)^3^, VSI (%) = viscera weight of fish (g) × 100/body weight of fish (g), and HSI (%) = liver weight of fish (g) × 100/body weight of fish (g).

### 2.4. Measurements of the Plasma Parameters

Blood was collected from the caudal veins of five anesthetized fish in each tank using heparinized syringes. For analysis, plasma samples were collected from each tank and pooled by dietary treatment groups. After centrifugation (2700×*g* at 4°C) for 10 min, the plasma was then extracted and stored as FUJI plain tube (0.5 mL) in a freezer at −70°C until further use. An automated chemistry system (Fuji Dri‐Chem NX500i; Fujifilm, Tokyo, Japan) was used to measure aspartate transaminase (AST), alanine transaminase (ALT), alkaline phosphatase (ALP), total bilirubin (T‐BIL), total cholesterol (T‐CHO), triglyceride (TG), total protein (TP), and albumin (ALB).

### 2.5. Measurements of the Biochemical Composition

The experimental feeds, 10 juveniles at the beginning of the experiment, and all live fish (≥23 fish), including those remaining after measuring for biological indices and plasma parameters, were homogenized for the biochemical composition analysis. The chemical composition of the samples was analyzed following the AOAC [29] methods. The moisture content of the experimental feeds and the whole‐body fish was determined in dry oven at 105°C for 6 and 24 h, respectively. The crude protein content was measured using a Kjeldahl method (Kjeltec 2100 Distillation Unit; Foss Tecator, Hoganas, Sweden). The crude lipid content was analyzed by the ether extraction method (Soxtec 2043 Fat Extraction System; Foss Tecator, Hoganas, Sweden). The ash content was measured using a muffle furnace at 550°C for 4 h. The gross energy of the experimental feeds was measured using a bomb calorimeter (Model 6100; Parr Instrument Co., Moline, IL, USA).

For AA analysis excluding tryptophan, the experimental feeds and the whole‐body fish were hydrolyzed with 6N HCl at 110°C for 24 h and analyzed via ion‐exchange chromatography using an AA analyzer (L‐8800 Auto‐analyzer; Hitachi, Tokyo, Japan). Tryptophan content was determined using high‐performance liquid chromatography (S1125 HPLC pump system; Sykam GmbH, Eresing, Germany). All EAAs, except for arginine, were present at relatively lower levels in DBM than in FM (Table 2). Among the non‐EAAs, alanine, glycine, and proline were present at relatively higher levels in DBM than in FM. As the level of dietary FM substitution with DBM increased, the contents of arginine, alanine, glycine, and proline increased, whereas those of all other AAs decreased.

**Table 2:** Amino acid profiles (% of the diet) of the experimental diets.

Amino acids	Ingredients		Experimental diets					
	FM	DBM	Con	DBM20	DBM40	DBM60	DBM80	DBM100
*Essential amino acids* (*EAAs*, *%*)								
Arginine	4.08	4.21	3.21	3.22	3.28	3.35	3.40	3.46
Histidine	1.73	1.24	1.47	1.37	1.33	1.31	1.27	1.23
Isoleucine	2.87	2.00	2.23	2.12	2.07	2.03	1.97	1.92
Leucine	5.31	3.98	4.69	4.31	4.23	4.18	4.07	3.85
Lysine	5.49	3.55	3.79	3.76	3.70	3.64	3.58	3.54
Phenylalanine	2.83	2.14	2.41	2.31	2.25	2.23	2.18	2.16
Threonine	3.09	2.25	2.33	2.22	2.15	2.11	2.07	2.05
Tryptophan	0.62	0.50	0.57	0.54	0.52	0.51	0.50	0.45
Valine	3.49	2.55	2.70	2.65	2.59	2.57	2.50	2.44
∑EAAs^a^	29.51	22.42	23.40	22.50	22.12	21.93	21.54	21.10
*Non-essential amino acids* (*NEAAs*, *%*)								
Alanine	4.39	4.42	3.14	3.19	3.21	3.37	3.41	3.45
Aspartic acid	6.34	4.74	4.90	4.75	4.69	4.63	4.53	4.24
Glutamic acid	8.88	7.86	8.01	7.72	7.40	7.39	7.37	7.05
Glycine	4.03	6.73	2.73	3.37	3.68	4.28	4.75	5.54
Proline	2.79	4.35	2.33	2.53	2.74	3.06	3.32	3.47
Serine	2.87	2.27	2.43	2.24	2.18	2.15	2.12	2.00
Tyrosine	2.15	1.41	1.82	1.71	1.67	1.53	1.40	1.30
∑NEAAs^b^	31.45	31.78	25.36	25.21	25.57	26.41	26.90	27.05

^a^∑EAAs: total essential amino acids.

^b^∑NEAAs: total non‐essential amino acids.

For FA analysis in the experimental feeds and the whole‐body fish were compared to known standards (37 component FAME mix CRM47885; Supelco, St. Louis, MO, USA). Lipids were extracted using a chloroform–methanol mixture (2:1, v/v) following the method by Folch et al. [30]. FA methyl esters were prepared by transesterification with 14% BF_3_‐MeOH (Sigma; St. Louis, MO, USA). The methylated samples were analyzed using gas chromatography (HP 6890; Agilent Technologies Inc., Santa Clara, CA, USA). equipped with an SP‐2560 capillary column (100 m × 0.25 mm inner diameter and 0.20 μm film thickness; Supelco, Bellefonte, PA, USA) and a flame ionization detector. FA profiles were identified by comparison with known FA standards (37‐component FAME mix CRM47885; Supelco, St. Louis, MO, USA). The total saturated FA (∑SFA) and monounsaturated FA (∑MUFA) were presented at comparatively higher than in DBM than those in FM, whereas arachidonic acid (ARA, 20:4n‐6) and ∑n‐3 HUFA including eicosapentaenoic acid (EPA, C20:5n‐3) and docosahexaenoic acid (DHA, C22:6n‐3) were comparatively lower in DBM than those in FM (Table 3). As the level of dietary FM substitution with DBM increased, ∑SFA and ∑MUFA increased, but ARA and ∑n‐3 HUFA decreased.

**Table 3:** Fatty acid profiles (% of total fatty acids) of the experimental diets.

Fatty acids	Ingredients		Experimental diets					
	FM	DBM	Con	DBM20	DBM40	DBM60	DBM80	DBM100
C12:0	0.06	0.19	0.03	0.04	0.05	0.07	0.09	0.10
C14:0	6.70	1.33	4.72	4.45	4.11	3.82	3.50	3.24
C16:0	23.32	29.00	15.33	16.54	17.72	18.98	20.40	21.02
C18:0	3.62	11.69	3.05	3.90	4.85	5.82	6.29	7.44
C20:0	0.11	0.03	0.40	0.13	0.10	0.08	0.07	0.03
C22:0	0.09	2.51	0.10	0.40	0.74	0.80	1.00	1.51
C24:0	1.29	0.15	0.90	0.88	0.81	0.72	0.51	0.45
∑SFA^a^	35.19	44.90	24.53	26.34	28.38	30.29	31.86	33.79
C14:1n‐5	0.06	0.08	0.04	0.04	0.05	0.08	0.09	0.10
C15:1n‐5	0.13	0.01	0.21	0.20	0.19	0.17	0.16	0.14
C16:1n‐7	9.12	4.13	5.28	5.22	5.19	5.07	4.92	4.76
C17:1n‐7	1.35	0.22	0.67	0.59	0.54	0.47	0.46	0.39
C18:1n‐9	14.52	44.91	15.65	18.46	21.51	24.97	27.67	31.30
C20:1n‐9	1.93	1.03	7.78	7.76	7.72	7.69	7.61	7.58
C22:1n‐9	0.16	0.06	0.20	0.19	0.16	0.15	0.11	0.09
C24:1n‐9	0.25	0.02	0.11	0.10	0.09	0.08	0.07	0.04
∑MUFA^b^	27.52	50.46	29.94	32.56	35.45	38.68	41.09	44.40
C18:2n‐6	5.27	2.99	19.35	15.81	13.45	10.81	8.72	5.78
C18:3n‐3	0.43	0.04	1.50	1.42	1.07	0.84	0.65	0.33
C18:3n‐6	0.32	0.19	0.44	0.39	0.35	0.30	0.24	0.20
C20:4n‐6	1.12	0.35	5.99	5.94	5.92	5.88	5.81	5.71
C20:5n‐3	15.54	0.14	9.26	8.70	7.60	6.46	5.61	4.36
C22:2n‐6	0.67	0.03	0.40	0.35	0.32	0.27	0.23	0.22
C22:6n‐3	10.46	0.04	5.62	5.52	4.70	3.80	2.83	2.25
∑n‐3 HUFA^c^	26.00	0.18	14.88	14.22	12.30	10.26	8.44	6.61
Unknown	3.48	0.86	2.97	2.97	2.76	2.67	2.96	2.96

^a^∑SFA, total saturated fatty acids.

^b^∑MUFA, total monounsaturated fatty acids.

^c^∑n‐3 HUFA, total n‐3 highly unsaturated fatty acids.

### 2.6. Economic Analysis of the Study

The economic conversion ratio (ECR) and economic profit index (EPI) were evaluated following the method described by Martínez‐Llorens et al. [31] (ECR [USD/kg] = FC [kg/fish]/WG [kg/fish] × diet price [USD/kg]) and EPI (USD/fish) = (final weight [kg/fish] × selling price of fish [USD/kg]) – (FC [kg/fish] × diet price [USD/kg]). The price of each ingredient was as follows: FM = USD 2.37/kg, DBM = USD 0.54/kg, fermented soybean meal = USD 1.04/kg, wheat flour = USD 0.50/kg, fish oil = USD 2.07/kg, soybean oil = USD 1.63/kg, vitamin premix = USD 7.56/kg, mineral premix = USD 6.07/kg, and choline = USD 1.30/kg. The price of each experimental feed was calculated by multiplying the proportional contribution level of each ingredient by its price per kg and then summing these values across all ingredients. The price of juvenile olive flounder was evaluated at USD 35.71/kg (1 USD per 28 g fish).

### 2.7. Statistical Analysis

All data were examined to verify the assumptions of normality and homogeneity of variance using the Shapiro–Wilk test and Levene’s test, respectively. Percentage data were subjected to arcsine transformation prior to statistical analysis. Significant differences were examined using one‐way ANOVA and Tukey’s post‐hoc test, with all analysis performed using SPSS version 24.0 (IBM Corp., Armonk, NY, USA). Differences were considered statistically significant at *p*  < 0.05. The analysis of orthogonal polynomial contrasts was determined to decide whether statistical differences were linear, quadratic, or cubic. Moreover, regression analysis was conducted to determine the best‐fitting model. The *p*‐value was used to evaluate the appropriate FM replacement level for DBM (dependent variable). Principal component analysis (PCA) was performed to identify major contributing factors or patterns among the AA and FA profiles of the whole‐body fish using SPSS version 24.0. To investigate the association between AA and FA profiles of the whole‐body fish, and the dietary treatments, the data were log_2_‐transformed and subjected to hierarchical clustering analysis through Pearson correlation using average linkage.

## 3. Results

### 3.1. Performance of the Experimental Fish

At the completion of the 50‐day feeding experiment, survival of olive flounder varied between 94.67% and 97.33%, but it was not significantly (*p* > 0.7) affected by dietary FM replacement with DBM (Table 4).

**Table 4:** Survival (%), weight gain (WG, g/fish), and specific growth rate (SGR, %/day) of olive flounder fed the experimental diets.

Parameters	Experimental diets							Orthogonal polynomial contrasts			Regression analysis			
	Con	DBM20	DBM40	DBM60	DBM80	DBM100	*p*‐Value	Linear	Quadratic	Cubic	Model	*p*‐Value	Adj. *R* ^2^	*Y*_max_
IBW (g/fish)	6.19 ± 0.027	6.29 ± 0.071	6.29 ± 0.071	6.37 ± 0.071	6.35 ± 0.071	6.32 ± 0.122	*p* > 0.7	—	—	—	—	—	—	—
FBW (g/fish)	28.12 ± 0.174	28.18 ± 0.782	27.49 ± 0.364	22.72 ± 0.527	16.19 ± 0.530	14.85 ± 0.531	*p* < 0.0001	—	—	—	—	—	—	—
Survival (%)	94.67 ± 1.333	97.33 ± 1.333	96.00 ± 2.309	94.67 ± 1.333	97.33 ± 1.333	96.00 ± 2.309	*p* > 0.7	0.6278	0.8382	0.4984	NR	—	—	—
WG (g/fish)	21.93 ± 0.151^a^	21.89 ± 0.741^a^	21.19 ± 0.400^a^	16.34 ± 0.516^b^	9.84 ± 0.554^c^	8.53 ± 0.452^c^	*p* < 0.0001	0.0001	0.0001	0.0001	*Y* = 0.00004259*X* ^3^ − 0.007713*X* ^2^ + 0.211515*X* + 21.5563	*p* < 0.0001	0.9566	15.77
SGR^1^ (%/day)	3.03 ± 0.006^a^	3.00 ± 0.045^a^	2.95 ± 0.042^a^	2.54 ± 0.047^b^	1.87 ± 0.075^c^	1.71 ± 0.051^c^	*p* < 0.0001	0.0001	0.0001	0.0008	*Y* = 0.000000033674*X* ^3^ − 0.000006457*X* ^2^ + 0.0001774*X* + 0.0298	*p* < 0.0001	0.9505	15.59

*Note:* Con, 60% fish meal (FM)‐based diet; DBM20, dietary 20% replacement of FM with DBM; DBM40, dietary 40% replacement of FM with DBM; DBM60, dietary 60% replacement of FM with DBM; DBM80, dietary 80% replacement of FM with DBM; DBM100, dietary 100% replacement of FM with DBM. Values (means of triplicate ± SE) in the same row sharing the same superscript letter are not significantly different (*p* > 0.05).

Abbreviations: Adj. *R* ^2^, adjusted *R* ^2^; FBW, final body weight; IBW, initial body weight; NR, no relationship.

^1^Specific growth rate (SGR, %/day) = [Ln final weight of fish (g) − Ln initial weight of fish (g)] × 100/days of feeding (50 days).

WG and SGR of fish fed the Con, DBM20, and DBM40 diets were significantly (*p* < 0.0001 for both) superior to those of fish fed the DBM60, DBM80, and DBM100 diets. In addition, WG and SGR of fish fed the DBM60 diet were also significantly (*p* < 0.05) higher than those of fish fed the DBM80 and DBM100 diets. Based on orthogonal polynomial contrast, WG and SGR of olive flounder exhibited linear (*p* < 0.0001 for both), quadratic (*p* < 0.0001 for both), and cubic (*p* < 0.0001 and *p* = 0.0008, respectively) models with increased DBM substitution levels for FM in the diet. According to regression analysis, cubic relationships were exhibited to be the most appropriate models between dietary increased DBM substitution levels for FM (X) and WG (*Y* = 0.00004259*X* ^3^ − 0.007713*X* ^2^ + 0.211515*X* + 21.5563, *p*  < 0.0001, Adj. *R* ^2^ = 0.9566, *Y*_max_ = *X* value of 15.77%), and SGR (*Y* = 0.000000033674*X* ^3^ − 0.000006457*X* ^2^ + 0.0001774*X* + 0.0298, *p*  < 0.0001, Adj. *R* ^2^ = 0.9505, *Y*_max_ = *X* value of 15.59%).

### 3.2. Feed Availability and Biological Indices of Fish

FC of olive flounder fed the Con, DBM20, and DBM40 feeds was significantly (*P* < 0.0001) elevated compared with that of fish fed the DBM60, DBM80, and DBM100 feeds (Table 5). In addition, FC of olive flounder fed the DBM60 feed was also statistically (*p* < 0.05) superior to that of fish fed the DBM80 and DBM100 feeds. In orthogonal polynomial contrasts, FC of olive flounder revealed linear (*p* < 0.0001), quadratic (*p* < 0.0001), and cubic (*p* < 0.0001) relationships with dietary DBM substitution levels for FM. Based on regression analysis, a cubic relationship was identified as the optimal model between dietary FM replacement with DBM (*X*) and FC (*Y* = 0.00004720*X* ^3^ − 0.008625*X* ^2^ + 0.275474*X* + 19.9037, *p*  < 0.0001, Adj. *R* ^2^ = 0.9615, *Y*_max_ = *X* value of 18.90%).

**Table 5:** Feed consumption (FC, g/fish), feed efficiency (FE), protein efficiency ratio (PER), protein retention (PR, %), condition factor (CF, g/cm^3^), viscerosomatic index (VSI, %), and hepatosomatic index (HSI, %) of olive flounder fed the experimental diets.

Parameters	Experimental diets							Orthogonal polynomial contrasts			Regression analysis			
	Con	DBM20	DBM40	DBM60	DBM80	DBM100	*p*‐Value	Linear	Quadratic	Cubic	Model	*p*‐Value	Adj. *R* ^2^	*Y*_max_
FC (g/fish)	20.26 ± 0.213^a^	21.25 ± 0.266^a^	20.96 ± 0.457^a^	16.14 ± 0.325^b^	9.94 ± 0.519^c^	8.73 ± 0.427^c^	*p* < 0.0001	0.0001	0.0001	0.0001	*Y* = 0.00004720*X* ^3^ − 0.008625*X* ^2^ + 0.275474*X* + 19.9037	*p* < 0.0001	0.9615	18.90
FE^1^	1.08 ± 0.013	1.03 ± 0.033	1.01 ± 0.030	1.02 ± 0.038	0.99 ± 0.020	0.98 ± 0.016	*p* > 0.1	0.0162	0.5264	0.5124	NR	—	—	—
PER^2^	2.07 ± 0.025	1.96 ± 0.062	1.93 ± 0.060	1.95 ± 0.072	1.89 ± 0.039	1.87 ± 0.027	*p* > 0.1	0.1475	0.5054	0.3837	NR	—	—	—
PR^3^ (%)	33.29 ± 0.481	32.20 ± 0.974	31.43 ± 1.118	31.98 ± 1.395	31.90 ± 0.526	33.06 ± 0.311	*p* > 0.6	0.8410	0.1121	0.9137	NR	—	—	—
CF^4^ (g/cm^3^)	1.01 ± 0.020^a^	0.94 ± 0.017^ab^	0.94 ± 0.013^ab^	0.90 ± 0.008^b^	0.88 ± 0.024^b^	0.73 ± 0.017^c^	*p* < 0.0001	0.0001	0.0158	0.0043	*Y* = − 0.0000009311*X* ^3^ + 0.00012*X* ^2^ − 0.005494*X* + 1.0127	*p* < 0.0001	0.8998	—
VSI^5^ (%)	6.20 ± 0.088	6.17 ± 0.096	6.32 ± 0.192	6.52 ± 0.138	6.38 ± 0.230	6.44 ± 0.044	*p* > 0.5	0.1229	0.6233	0.5976	NR	—	—	—
HSI^6^ (%)	2.11 ± 0.070	2.19 ± 0.059	2.17 ± 0.051	2.06 ± 0.065	2.12 ± 0.082	2.12 ± 0.056	*p* > 0.7	0.5626	0.9344	0.2999	NR	—	—	—

*Note:* Values (means of triplicate ± SE) in the same row sharing the same superscript letter are not significantly different (*p* > 0.05).

Abbreviations: Adj. *R* ^2^, adjusted *R* ^2^; Con: 60% fish meal (FM)‐based diet; DBM20: dietary 20% replacement of FM with DBM, DBM40: dietary 40% replacement of FM with DBM; DBM60: dietary 60% replacement of FM with DBM; DBM80: dietary 80% replacement of FM with DBM; DBM100: dietary 100% replacement of FM with DBM; NR, no relationship.

^1^Feed efficiency (FE) = [total final weight of fish (g) − total initial weight of fish (g) + total weight of dead fish (g)]/total feed consumption (g).

^2^Protein efficiency ratio (PER) = weight gain of fish (g/fish)/protein consumption of fish (g/fish).

^3^Protein retention (PR, %) = protein gain of fish (g/fish) × 100/protein consumption of fish (g/fish).

^4^Condition factor (CF, g/cm^3^) = body weight of fish (g) × 100/total length of fish (cm)^3^.

^5^Viscerosomatic index (VSI, %) = viscera weight of fish (g) × 100/body weight of fish (g).

^6^Hepatosomatic index (HSI, %) = liver weight of fish (g) × 100/body weight of fish (g).

FE ranged from 0.98 to 1.08, PER ranged from 1.87 to 2.07, and PR ranged from 31.43% to 33.29%, respectively. However, no significant differences (*p* > 0.1, *p*  > 0.1, and *p*  > 0.6) were found in FE, PER, and PR among dietary FM replacements with DBM.

CF of olive flounder supplied with the Con diet was significantly (*p* < 0.0001) elevated compared with that of fish supplied with the DBM60, DBM80, and DBM100 diets, but was comparable to that of fish supplied with the DBM20 and DBM40 diets. In orthogonal polynomial contrasts, CF of olive flounder revealed linear (*p* < 0.0001), quadratic (*p* = 0.0158), and cubic (*p* = 0.0043) relationships with dietary elevated DBM substitution levels for FM. In regression analysis, a cubic relationship was shown to be the most appropriate model between increased dietary FM replacements with DBM (*X*) and CF (*Y* = − 0.0000009311*X* ^3^ + 0.00012*X* ^2^ − 0.005494*X* + 1.0127, *p*  < 0.0001, Adj. *R* ^2^ = 0.8998). However, VSI (6.17%−6.52%) and HSI (2.06%−2.19%) of fish were not significantly (*p* > 0.5 and *p*  > 0.7, respectively) altered by dietary treatments.

### 3.3. Plasma Parameters of Fish

Plasma AST, ALT, ALP, T‐BIL, T‐CHO, TG, TP, and ALB ranged from 48.6–52.6 U/L, 11.1–11.5 U/L, 119.7–141.0 U/L, 0.3–0.4 mg/dL, 268.8–292.4 mg/dL, 404.8–408.7 mg/dL, 4.4–4.7 g/dL, and 0.9–1.0 g/dL, respectively (Table 6). None of these parameters showed significant (*p* > 0.05 for all) differences among dietary treatments.

**Table 6:** Plasma parameters of olive flounder fed the experimental diets replacing various levels of fish meal with duck by‐product meal.

Parameters	Experimental diets						*p*‐Value	Orthogonal polynomial contrasts			Regression analysis		
	Con	DBM20	DBM40	DBM60	DBM80	DBM100		Linear	Quadratic	Cubic	Model	*p*‐Value	Adj. *R* ^2^
AST (U/L)	49.9 ± 3.28	52.0 ± 2.83	49.8 ± 4.15	48.6 ± 3.29	50.8 ± 3.01	52.6 ± 0.78	*p* > 0.9	0.7478	0.5772	0.5276	NR	—	—
ALT (U/L)	11.5 ± 0.19	11.3 ± 0.51	11.3 ± 0.33	11.1 ± 0.59	11.1 ± 0.56	11.2 ± 0.11	*p* > 0.9	0.5481	0.7436	0.8429	NR	—	—
ALP (U/L)	129.8 ± 6.65	134.0 ± 12.31	119.7 ± 9.53	133.3 ± 11.18	141.0 ± 21.32	132.1 ± 20.44	*p* > 0.9	0.7117	0.8699	0.6477	NR	—	—
T‐BIL (mg/dL)	0.3 ± 0.07	0.4 ± 0.10	0.4 ± 0.07	0.4 ± 0.07	0.4 ± 0.12	0.4 ± 0.03	*p* > 0.9	0.6625	0.6901	0.7088	NR	—	—
T‐CHO (mg/dL)	273.7 ± 12.00	268.8 ± 17.73	269.2 ± 19.13	290.0 ± 17.02	270.9 ± 14.33	292.4 ± 25.44	*p* > 0.8	0.4399	0.7504	0.9871	NR	—	—
TG (mg/dL)	405.6 ± 3.26	408.7 ± 7.34	406.7 ± 5.84	408.0 ± 2.40	404.8 ± 5.08	406.2 ± 2.89	*p* > 0.9	0.8646	0.7691	0.7024	NR	—	—
TP (g/dL)	4.5 ± 0.19	4.4 ± 0.30	4.5 ± 0.32	4.4 ± 0.29	4.5 ± 0.34	4.7 ± 0.18	*p* > 0.9	0.5247	0.5865	0.8793	NR	—	—
ALB (g/dL)	1.0 ± 0.09	0.9 ± 0.08	1.0 ± 0.07	0.9 ± 0.08	0.9 ± 0.13	1.0 ± 0.10	*p* > 0.9	0.8360	0.5815	0.7311	NR	—	—

*Note:* Values (means of triplicate ± SE) are presented.

Abbreviations: Adj. *R* ^2^, adjusted *R* ^2^; ALB, albumin; ALP, alkaline phosphatase; ALT, alanine aminotransferase; AST, aspartate aminotransferase; Con: 60% fish meal (FM)‐based diet; DBM20, dietary 20% replacement of FM with DBM; DBM40, dietary 40% replacement of FM with DBM; DBM60, dietary 60% replacement of FM with DBM; DBM80, dietary 80% replacement of FM with DBM; DBM100, dietary 100% replacement of FM with DBM; NR, no relationship; T‐BIL, total bilirubin; T‐CHO, total cholesterol; TG, triglyceride; TP, total protein.

### 3.4. Chemical Composition of the Whole‐Body Fish

Moisture content of the whole‐body fish ranged from 76.8% to 77.7%, crude protein content ranged from 15.3% to 15.7%, crude lipid content ranged from 2.9% to 3.3%, and ash content ranged from 2.7% to 3.0% (Table 7). None of these measurements were significantly (*p* > 0.1, *p*  > 0.07, *p*  > 0.1, and *p*  > 0.3, respectively) affected by dietary FM substitutions with DBM.

**Table 7:** Chemical composition (% of wet weight) of the whole‐body olive flounder fed the experimental diets.

Parameters	Experimental diets							Orthogonal polynomial contrasts			Regression analysis		
	Con	DBM20	DBM40	DBM60	DBM80	DBM100	*p*‐Value	Linear	Quadratic	Cubic	Model	*p*‐value	Adj. R^2^
Moisture	77.2 ± 0.08	76.9 ± 0.31	76.8 ± 0.20	77.3 ± 0.34	77.6 ± 0.32	77.7 ± 0.24	*p* > 0.1	0.0456	0.1498	0.2232	NR	—	—
Crude protein	15.4 ± 0.10	15.6 ± 0.05	15.5 ± 0.06	15.4 ± 0.11	15.3 ± 0.08	15.7 ± 0.09	*p* > 0.07	0.6288	0.5257	0.0046	NR	—	—
Crude lipid	3.2 ± 0.10	3.3 ± 0.15	2.9 ± 0.10	2.9 ± 0.12	3.1 ± 0.03	3.1 ± 0.11	*p* > 0.1	0.2322	0.0725	0.4775	NR	—	—
Ash	2.8 ± 0.13	2.7 ± 0.06	2.8 ± 0.09	2.9 ± 0.05	2.7 ± 0.15	3.0 ± 0.12	*p* > 0.3	0.2002	0.2323	0.5220	NR	—	—

*Note:* Values (means of triplicate ± SE) are presented.

Abbreviations: Adj. *R* ^2^, adjusted *R* ^2^; Con, 60% fish meal (FM)‐based diet; DBM20, dietary 20% replacement of FM with DBM; DBM40, dietary 40% replacement of FM with DBM; DBM60, dietary 60% replacement of FM with DBM; DBM80, dietary 80% replacement of FM with DBM; DBM100, dietary 100% replacement of FM with DBM; NR, no relationship.

### 3.5. AA Profiles of the Whole‐Body Fish

Isoleucine of the whole‐body fish fed the Con diet was significantly (*p* < 0.0002) higher than that of fish fed the DBM40, DBM60, DBM80, and DBM100 diets (Table 8). Tryptophan of the whole‐body fish fed the Con diet was significantly (*p* < 0.04) higher than that of fish fed the DBM100 diet. Glycine of the whole‐body fish fed the DBM100 diet was significantly (*p* < 0.0001) higher than that of fish fed the Con, DBM20, and DBM40 diets. Proline of the whole‐body fish fed the DBM100 diet was significantly (*p* < 0.002) higher than that of fish fed the Con and DBM20 diets.

**Table 8:** Amino acid profiles (% of total amino acid) of the whole‐body olive flounder fed the experimental diets.

Amino acids	Experimental diets						*p*‐Value	Orthogonal polynomial contrasts			Regression analysis		
	Con	DBM20	DBM40	DBM60	DBM80	DBM100		Linear	Quadratic	Cubic	Model	*p*‐Value	Adj. *R* ^2^
*Essential amino acids* (*EAAs*, *%*)													
Arginine	7.08 ± 0.283	7.37 ± 0.328	7.57 ± 0.347	7.91 ± 0.526	8.23 ± 0.441	8.71 ± 0.361	*p* > 0.1	0.0053	0.6966	0.8834	NR	—	—
Histidine	2.15 ± 0.105	1.99 ± 0.275	1.92 ± 0.242	1.82 ± 0.170	1.57 ± 0.194	1.56 ± 0.245	*p* > 0.3	0.0317	0.9791	0.8856	NR	—	—
Isoleucine	4.27 ± 0.046^a^	4.08 ± 0.127^ab^	3.95 ± 0.028^bc^	3.86 ± 0.050^bc^	3.71 ± 0.035^c^	3.69 ± 0.024^c^	*p* < 0.0002	0.0001	0.2068	0.8776	*Y* = 0.00003404*X* ^2^ – 0.009248*X* + 4.2182	*p* < 0.0001	0.8131
Leucine	8.13 ± 0.038	7.90 ± 0.137	7.76 ± 0.246	7.71 ± 0.276	7.77 ± 0.112	7.43 ± 0.162	*p* > 0.2	0.0232	0.8847	0.3426	NR	—	—
Lysine	9.24 ± 0.080	9.15 ± 0.258	9.17 ± 0.217	9.01 ± 0.185	8.49 ± 0.288	8.44 ± 0.294	*p* > 0.1	0.0082	0.3903	0.6810	NR	—	—
Phenylalanine	4.31 ± 0.149	4.22 ± 0.304	4.22 ± 0.290	4.10 ± 0.137	3.96 ± 0.173	4.00 ± 0.146	*p* > 0.8	0.1926	0.9659	0.7958	NR	—	—
Threonine	4.97 ± 0.315	4.87 ± 0.223	4.68 ± 0.249	4.55 ± 0.163	4.52 ± 0.187	4.44 ± 0.255	*p* > 0.5	0.0789	0.7499	0.9121	NR	—	—
Tryptophan	0.69 ± 0.051^a^	0.55 ± 0.084^ab^	0.49 ± 0.068^ab^	0.45 ± 0.087^ab^	0.36 ± 0.050^ab^	0.31 ± 0.077^b^	*p* < 0.04	0.0012	0.6199	0.7316	*Y* = –0.003573*X* + 0.6522	*p* < 0.0002	0.5591
Valine	5.06 ± 0.339	4.90 ± 0.307	4.68 ± 0.223	4.55 ± 0.266	4.37 ± 0.318	3.98 ± 0.247	*p* > 0.1	0.0116	0.7112	0.7618	NR	—	—
*Non-essential amino acids* (*NEAAs*, *%*)													
Alanine	7.29 ± 0.341	7.69 ± 0.201	7.93 ± 0.307	8.23 ± 0.252	8.48 ± 0.322	8.82 ± 0.468	*p* > 0.06	0.0026	0.9234	0.8476	NR	—	—
Aspartic acid	10.52 ± 0.457	9.32 ± 0.265	9.34 ± 0.258	9.33 ± 0.215	9.40 ± 0.313	9.49 ± 0.374	*p* > 0.1	0.0949	0.0444	0.2194	NR	—	—
Glutamic acid	15.91 ± 0.165	15.30 ± 0.155	15.15 ± 0.222	15.23 ± 0.370	15.42 ± 0.389	15.21 ± 0.282	*p* > 0.4	0.2105	0.2090	0.2370	NR	—	—
Glycine	7.85 ± 0.040^d^	9.73 ± 0.203^c^	10.12 ± 0.065^bc^	10.42 ± 0.085^abc^	10.77 ± 0.198^ab^	11.15 ± 0.345^a^	*p* < 0.0001	0.0001	0.0008	0.0079	*Y* = 0.000009292*X* ^3^ – 0.001736*X* ^2^ + 0.113668*X* + 7.9028	*p* < 0.0001	0.9164
Proline	4.69 ± 0.039^c^	5.60 ± 0.177^bc^	6.00 ± 0.208^ab^	6.17 ± 0.274^ab^	6.43 ± 0.181^ab^	6.60 ± 0.211^a^	*p* < 0.002	0.0001	0.0349	0.2642	*Y* = 0.000003551X^3^ – 0.000723X^2^ + 0.055865*X* + 4.7055	*p* < 0.0001	0.8023
Serine	4.58 ± 0.227	4.25 ± 0.180	4.13 ± 0.194	3.98 ± 0.212	3.91 ± 0.125	3.79 ± 0.299	*p* > 0.1	0.0139	0.5188	0.7308	NR	—	—
Tyrosine	3.26 ± 0.266	3.07 ± 0.129	2.88 ± 0.209	2.69 ± 0.225	2.62 ± 0.140	2.36 ± 0.146	*p* > 0.06	0.0028	0.9363	0.8523	NR	—	—

*Note:* Values (means of triplicate ± SE) in the same row sharing the same superscript letter are not significantly different (*p* > 0.05).

Abbreviations: Adj. *R* ^2^, adjusted *R* ^2^; NR, no relationship; Con, 60% fish meal (FM)‐based diet; DBM20, dietary 20% replacement of FM with DBM; DBM40, dietary 40% replacement of FM with DBM; DBM60, dietary 60% replacement of FM with DBM; DBM80, dietary 80% replacement of FM with DBM; DBM100, dietary 100% replacement of FM with DBM.

In orthogonal polynomial contrasts, isoleucine and tryptophan of the whole‐body fish exhibited linear (*p* < 0.0001 and *p*  < 0.0012, respectively) relationships with increased DBM substitution levels for FM in the diets. Glycine of the whole‐body fish exhibited linear (*p* < 0.0001), quadratic (*p* < 0.0008), and cubic (*p* < 0.0079) relationships, whereas proline of the whole‐body fish exhibited linear (*p* < 0.0001) and quadratic (*p* < 0.0349) relationships with increased DBM substitution levels for FM in the diets. In regression analysis, a quadratic relationship was identified as the best fit between increased dietary FM replacement with DBM (X) and isoleucine (*Y* = 0.00003404*X* ^2^ – 0.009248*X* + 4.2182, *p*  < 0.0001, Adj. *R* ^2^ = 0.8131) of the whole‐body fish. A linear relationship was identified as the best fit between increased dietary FM replacements with DBM (*X*) and tryptophan (*Y* = –0.003573*X* + 0.6522, *p*  < 0.0002, Adj. *R* ^2^ = 0.5591) of the whole‐body fish. A cubic relationship was identified as the best fit between increased dietary FM replacements with DBM (*X*) and glycine (*Y* = 0.000009292*X* ^3^ – 0.001736*X* ^2^ + 0.113668*X* + 7.9028, *p*  < 0.0001, Adj. *R* ^2^ = 0.9164) and proline (*Y* = 0.000003551*X* ^3^ – 0.000723*X* ^2^ + 0.055865*X* + 4.7055, *p*  < 0.0001, Adj. *R* ^2^ = 0.8023) of the whole‐body fish.

**Figure 1:**
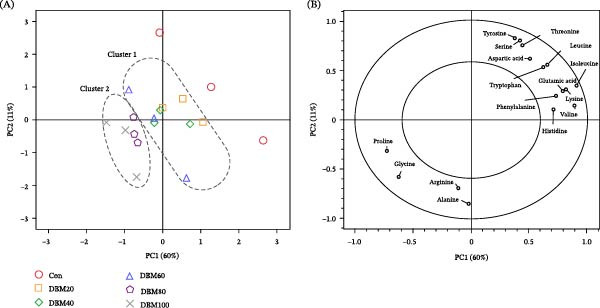
Principal component analysis (PCA) of the whole‐body amino acid profiles of olive flounder fed the experimental diets: (A) score plot and (B) correlation loading plot. Con: 60% fish meal (FM)‐based diet; DBM20: dietary 20% replacement of FM with DBM: DBM40: dietary 40% replacement of FM with DBM; DBM60: dietary 60% replacement of FM with DBM; DBM80: dietary 80% replacement of FM with DBM; DBM100: dietary 100% replacement of FM with DBM.

One distinct separation (the Con diet) and two clusters clearly classified AA profiles of the whole‐body fish in the PCA score plot (Figure 1A). The first two principal components account for 71% of the total variation (PC1:60% and PC2:11%). Cluster 1 comprised the whole‐body fish provided with the DBM20, DBM40, and DBM60 diets, and cluster 2 included the whole‐body fish provided with the DBM80 and DBM100 diets. In correlation loading plot, isoleucine, valine, lysine, glutamic acid, phenylalanine, histidine, leucine, and tryptophan were the primary variables associated with PC1 (Figure 1B), while tyrosine, serine, threonine, and aspartic acid were the most important variables in PC2. Histidine, isoleucine, leucine, lysine, phenylalanine, threonine, tryptophan, valine, aspartic acid, glutamic acid, serine, and tyrosine were closely associated with the AA profiles of the whole‐body fish supplied with the Con feed, whereas arginine, alanine, glycine, and proline were closely associated with the AA profiles of the whole‐body fish provided with the DBM80 and DBM100 feeds.

### 3.6. FA Profiles of the Whole‐Body Fish

Increasing FM replacement levels with DBM in diets resulted in higher ∑SFA and total ∑MUFA of the whole‐body fish but decreased ∑n‐3 HUFA and arachidonic acid (Table 9). In orthogonal polynomial contrasts, ∑SFA of the whole‐body fish exhibited linear (*p* < 0.0001) and quadratic (*p* = 0.0133) relationships with elevated DBM replacement levels for FM in the feeds, ∑MUFA of the whole‐body fish showed linear (*p* < 0.0001), quadratic (*p* = 0.0011), and cubic (*p* < 0.0001) relationships, and ∑n‐3 HUFA exhibited linear (*p* < 0.0001) and cubic (*p* = 0.0064) trends. In regression analysis, a quadratic relationship was identified as the best model between dietary increased DBM replacement levels for FM (X) and the whole‐body ∑SFA (*Y* = 0.000177*X* ^2^ + 0.017020*X* + 24.7669, *p*  < 0.0001, Adj. *R* ^2^ = 0.9605). In contrast, linear model exhibited the best fit between dietary increased DBM replacement levels for FM (*X*) and the whole‐body ∑MUFA (*Y* = 0.099481*X* + 30.8365, *p*  < 0.0001, Adj. *R* ^2^ = 0.9576), and ∑n‐3 HUFA (*Y* = − 0.020095*X* + 17.7858, *p*  < 0.0001, Adj. *R* ^2^ = 0.9002). In addition, linear relationships showed the best fit model between dietary elevated DBM replacement levels for FM (*X*) and the whole‐body ARA (*Y* = − 0.004305*X* + 5.614127, *p*  < 0.0001, Adj. *R* ^2^ = 0.6480) or EPA (*Y* = − 0.017571*X* + 9.4019, *p*  < 0.0001, Adj. *R* ^2^ = 0.9130) in regression analysis.

**Table 9:** Fatty acid profiles (% of total fatty acids) of the whole‐body olive flounder fed the experimental diets.

Fatty acids	Experimental diets							Orthogonal polynomial contrasts			Regression analysis		
	Con	DBM20	DBM40	DBM60	DBM80	DBM100	*p*‐Value	Linear	Quadratic	Cubic	Model	*P*‐value	Adj. *R* ^2^
C12:0	0.04 ± 0.003^d^	0.05 ± 0.003^cd^	0.06 ± 0.006^c^	0.07 ± 0.003^bc^	0.09 ± 0.003^ab^	0.10 ± 0.006^a^	*p* < 0.0001	0.0001	0.2935	0.8654	*Y* = 0.000633*X* + 0.03444	*p* < 0.0001	0.9074
C14:0	4.49 ± 0.075^a^	4.21 ± 0.064^ab^	4.19 ± 0.059^b^	4.10 ± 0.039^b^	4.07 ± 0.077^b^	3.61 ± 0.058^c^	*p* < 0.0001	0.0001	0.1512	0.0037	*Y* = − 0.000003526*X* ^3^ + 0.000489*X* ^2^ − 0.022384*X* + 4.4939	*p* < 0.0001	0.8588
C16:0	14.60 ± 0.078^e^	16.35 ± 0.075^d^	16.66 ± 0.105^d^	17.54 ± 0.075^c^	18.57 ± 0.048^b^	20.02 ± 0.046^a^	*p* < 0.0001	0.0001	0.0629	0.0001	*Y* = 0.000009263*X* ^3^ − 0.001327*X* ^2^ + 0.094061*X* + 14.6751	*p* < 0.0001	0.9853
C18:0	2.65 ± 0.121	2.66 ± 0.072	2.67 ± 0.012	2.70 ± 0.049	2.71 ± 0.025	2.73 ± 0.041	*p* > 0.9	0.3202	0.9558	0.8976	NR	—	—
C20:0	1.63 ± 0.052^a^	0.58 ± 0.033^b^	0.54 ± 0.036^b^	0.50 ± 0.040^bc^	0.47 ± 0.017^bc^	0.33 ± 0.068^c^	*p* < 0.0001	0.0001	0.0001	0.0001	*Y* = − 0.000006454*X* ^3^ + 0.001172*X* ^2^ − 0.065578*X* + 1.5900	*p* < 0.0001	0.9393
C22:0	0.44 ± 0.026^b^	0.64 ± 0.034^a^	0.70 ± 0.032^a^	0.72 ± 0.017^a^	0.74 ± 0.035^a^	0.75 ± 0.042^a^	*p* < 0.0002	0.0001	0.0031	0.0880	*Y* = − 0.00004836*X* ^2^ + 0.007522*X* + 0.4667	*p* < 0.0001	0.7707
C24:0	0.89 ± 0.041	0.82 ± 0.101	0.80 ± 0.035	0.77 ± 0.052	0.72 ± 0.024	0.65 ± 0.027	*p* > 0.1	0.0061	0.8309	0.6049	NR	—	—
∑SFA^1^	24.73 ± 0.151^e^	25.30 ± 0.056^de^	25.61 ± 0.171^d^	26.39 ± 0.169^c^	27.37 ± 0.049^b^	28.20 ± 0.218^a^	*p* < 0.0001	0.0001	0.0133	0.8807	*Y* = 0.000177*X* ^2^ + 0.017020*X* + 24.7669	*p* < 0.0001	0.9605
C14:1n‐5	0.22 ± 0.017^b^	0.22 ± 0.026^b^	0.24 ± 0.023^ab^	0.25 ± 0.015^ab^	0.28 ± 0.023^ab^	0.33 ± 0.026^a^	*p* < 0.03	0.0019	0.2270	0.8958	*Y* = 0.001057*X* + 0.2038	*p* < 0.0006	0.5016
C15:1n‐5	0.12 ± 0.009^a^	0.07 ± 0.015^ab^	0.06 ± 0.009^b^	0.05 ± 0.009^b^	0.03 ± 0.012^b^	0.02 ± 0.009^b^	*p* < 0.005	0.0001	0.1101	0.2438	*Y* = − 0.000843*X* + 0.0993	*p* < 0.0001	0.6866
C16:1n‐7	4.90 ± 0.046^a^	4.74 ± 0.086^a^	4.71 ± 0.035^a^	4.69 ± 0.049^ab^	4.46 ± 0.051^bc^	4.36 ± 0.035^c^	*p* < 0.0002	0.0001	0.3016	0.3553	*Y* = − 0.005100*X* + 4.8977	*p* < 0.0001	0.7733
C17:1n‐7	0.87 ± 0.020	0.85 ± 0.026	0.84 ± 0.012	0.82 ± 0.033	0.81 ± 0.023	0.79 ± 0.009	*p* > 0.2	0.0168	0.8721	0.7091	NR	—	—
C18:1n‐9	18.04 ± 0.274^f^	21.16 ± 0.099^e^	24.66 ± 0.065^d^	25.56 ± 0.133^c^	26.82 ± 0.130^b^	30.29 ± 0.063^a^	*p* < 0.0001	0.0001	0.0002	0.0001	*Y* = 0.00002081*X* ^3^ − 0.003444*X* ^2^ + 0.259363*X* + 17.8391	*p* < 0.0001	0.9832
C20:1n‐9	5.92 ± 0.047^a^	5.37 ± 0.072^b^	5.23 ± 0.049^b^	5.20 ± 0.058^b^	5.19 ± 0.075^b^	5.17 ± 0.046^b^	*p* < 0.0001	0.0001	0.0001	0.0111	*Y* = 0.000002755*X* ^3^ + 0.000555*X* ^2^ − 0.035466*X* + 5.9134	*p* < 0.0001	0.8844
C22:1n‐9	0.15 ± 0.023	0.13 ± 0.017	0.12 ± 0.027	0.10 ± 0.028	0.06 ± 0.019	0.05 ± 0.022	*p* > 0.05	0.0020	0.7928	0.8081	NR	—	—
C24:1n‐9	0.19 ± 0.023	0.17 ± 0.026	0.16 ± 0.020	0.15 ± 0.025	0.14 ± 0.039	0.12 ± 0.060	*p* > 0.8	0.1645	0.9758	0.7775	NR	—	—
∑MUFA^2^	30.41 ± 0.090^f^	32.70 ± 0.126^e^	36.01 ± 0.057^d^	36.83 ± 0.123^c^	37.79 ± 0.094^b^	41.12 ± 0.131^a^	*p* < 0.0001	0.0001	0.0011	0.0001	*Y* = 0.099481*X* + 30.8365	*p* < 0.0001	0.9576
C18:2n‐6	18.24 ± 0.185^a^	17.18 ± 0.036^b^	14.81 ± 0.084^c^	13.46 ± 0.122^d^	11.44 ± 0.049^e^	8.46 ± 0.155^f^	*p* < 0.0001	0.0001	0.0001	0.0581	*Y* = − 0.000365*X* ^2^ − 0.059927*X* + 18.2659	*p* < 0.0001	0.9899
C18:3n‐3	1.57 ± 0.026^a^	1.23 ± 0.058^b^	0.82 ± 0.045^c^	0.81 ± 0.012^c^	0.66 ± 0.022^c^	0.39 ± 0.013^d^	*p* < 0.0001	0.0001	0.0008	0.0013	*Y* = 0.000002195*X* ^3^ + 0.000391*X* ^2^ − 0.028980*X* + 1.5887	*p* < 0.0001	0.9582
C18:3n‐6	0.21 ± 0.017	0.20 ± 0.022	0.19 ± 0.025	0.18 ± 0.015	0.13 ± 0.018	0.12 ± 0.024	*p* > 0.05	0.0024	0.4801	0.6956	NR	—	—
C20:4n‐6	5.64 ± 0.069^a^	5.59 ± 0.044^a^	5.36 ± 0.075^ab^	5.29 ± 0.059^b^	5.27 ± 0.035^b^	5.24 ± 0.073^b^	*p* < 0.002	0.0001	0.1039	0.5642	*Y* = − 0.004305*X* + 5.614127	*p* < 0.0001	0.6480
C20:5n‐3	9.66 ± 0.075^a^	8.87 ± 0.066^b^	8.51 ± 0.082^c^	8.35 ± 0.037^c^	8.02 ± 0.035^d^	7.73 ± 0.071^d^	*p* < 0.0001	0.0001	0.0007	0.0034	*Y* = − 0.017571*X* + 9.4019	*p* < 0.0001	0.9130
C22:2n‐6	0.35 ± 0.040	0.32 ± 0.055	0.31 ± 0.030	0.29 ± 0.026	0.28 ± 0.031	0.26 ± 0.022	*p* > 0.5	0.0639	0.8890	0.8488	NR	—	—
C22:6n‐3	8.33 ± 0.075	8.32 ± 0.111	8.30 ± 0.102	8.29 ± 0.123	8.22 ± 0.032	8.13 ± 0.052	*p* > 0.5	0.0918	0.4951	0.8396	NR	—	—
∑n‐3 HUFA^3^	17.99 ± 0.150^a^	17.19 ± 0.154^b^	16.80 ± 0.039^bc^	16.64 ± 0.135^c^	16.35 ± 0.040^c^	15.71 ± 0.069^d^	*p* < 0.0001	0.0001	0.2550	0.0064	*Y* = − 0.020095*X* + 17.7858	*p* < 0.0001	0.9002
Unknown	0.85 ± 0.180	0.30 ± 0.064	0.08 ± 0.043	0.12 ± 0.026	0.81 ± 0.081	0.34 ± 0.224	—	—	—	—	—	—	—

*Note:* Values (means of triplicate ± SE) in the same row sharing the same superscript letter are not significantly different (*p* > 0.05).

Abbreviations: Adj. *R* ^2^, adjusted *R* ^2^; Con, 60% fish meal (FM)‐based diet; DBM20, dietary 20% replacement of FM with DBM; DBM40, dietary 40% replacement of FM with DBM; DBM60, dietary 60% replacement of FM with DBM; DBM80, dietary 80% replacement of FM with DBM; DBM100, dietary 100% replacement of FM with DBM; NR, no relationship.

^1^∑SFA, total saturated fatty acids.

^2^∑MUFA, total monounsaturated fatty acids.

^3^∑n‐3 HUFA, total n‐3 highly unsaturated fatty acids.

**Figure 2:**
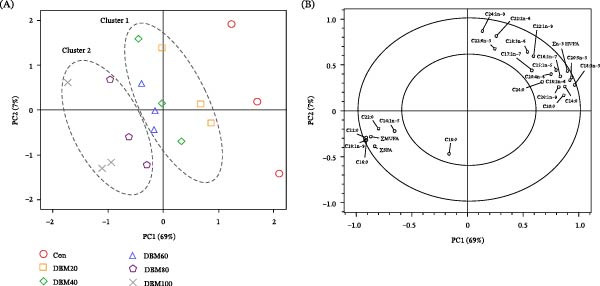
Principal component analysis (PCA) of the whole‐body fatty acid profiles of olive flounder fed the experimental diets: (A) score plot and (B) correlation loading plot. Con: 60% fish meal (FM)‐based diet; DBM20: dietary 20% replacement of FM with DBM: DBM40: dietary 40% replacement of FM with DBM; DBM60: dietary 60% replacement of FM with DBM; DBM80: dietary 80% replacement of FM with DBM; DBM100: dietary 100% replacement of FM with DBM; ∑SFA: total saturated fatty acids; ∑MUFA: total monounsaturated fatty acids; ∑n‐3 HUFA: total n‐3 highly unsaturated fatty acids.

The FA profiles of whole‐body fish were distinctly grouped into one distinct separation (the Con diet) and two clusters (Figure 2A). The first two principal components accounted for 76% of the total variation in the PCA model (PC1:69% and PC2:7%). Cluster 1 comprised the whole‐body fish provided with the DBM20, DBM40 and DBM60 diets, and cluster 2 included the whole‐body fish provided with the DBM80 and DBM100 diets. In correlation loading plot, α‐Linolenic acid (ALA, C18:3n‐3), EPA, linoleic acid (C18:2n‐6), ∑n‐3 HUFA, myristic acid (C14:0), arachidic acid (C20:0), palmitoleic acid (C16:1n‐7), eicosenoic acid (C20:1n‐9), pentadecenoic acid (C15:1n‐5), ARA, and lignoceric acid (C24:0) were the primary variables associated with PC1 (Figure 2B). Tetracosenoic acid (C24:1n‐9), docosadienoic acid (C22:2n‐6), DHA, and γ‐Linolenic acid (C18:3n‐6) were the most important variables in PC2. The ∑n‐3 HUFA including EPA and DHA, myristic acid, arachidic acid, lignoceric acid, pentadecenoic acid, palmitoleic acid, margaroleic acid (C17:1n‐7), eicosenoic acid, erucic acid (C22:1n‐9), tetracosenoic acid, linoleic acid, γ‐linolenic acid, ARA, docosadienoic acid, and α‐linolenic acid were closely related with the FA profiles of the whole‐body fish fed the Con diet, whereas lauric acid (C12:0), palmitic acid (C16:0), behenic acid (C22:0), ∑SFA, myristoleic acid (C14:1n‐5), oleic acid (C18:1n‐9), and ∑MUFA were strongly related to the FA profiles of the whole‐body fish fed the DBM80 and DBM100 diets.

### 3.7. Economic Analysis of the Experiment

The price of the experimental diets decreased with elevated dietary FM substitutions with DBM (Table 10). The ECR of the Con and DBM20 diets was significantly (*p* < 0.0001) higher than that of the DBM40, DBM60, DBM80, and DBM100 diets. In orthogonal polynomial contrasts, ECR revealed linear (*p* < 0.0001) relationship with dietary increased FM substitution levels for DBM. In regression analysis, a cubic relationship was shown to be the most appropriate model between increasing dietary FM replacement by DBM (*X*) and ECR (*Y* = 0.0000005015*X* ^3^ − 0.00009666X^2^ − 0.004942*X* + 1.2912, *p*  < 0.0001, Adj. *R* ^2^ = 0.9718). The EPI of the Con, DBM20, and DBM40 feeds was statistically (*p* < 0.0001) higher than that of the DBM60, DBM80, and DBM100 feeds. In orthogonal polynomial contrasts, EPI revealed linear (*p* < 0.0001), quadratic (*p* < 0.0001), and cubic (*p* = 0.0003) relationships with dietary increased DBM substitution levels for FM. In regression analysis, a cubic relationship was shown to be the best model between dietary elevated DBM replacements for FM (*X*) and EPI (*Y* = 0.000001431*X* ^3^ − 0.000263*X* ^2^ + 0.007631*X* + 0.9370, *p*  < 0.0001, Adj. *R* ^2^ = 0.9490, *Y*_max_ = *X* value of 16.81%).

**Table 10:** Economic parameters (diet price [USD/kg], economic conversion ratio [ECR, USD/kg], and economic profit index [EPI, USD/fish]) of the feeding experiment.

Parameters	Experimental diets							Orthogonal polynomial contrasts			Regression analysis			
	Con	DBM20	DBM40	DBM60	DBM80	DBM100	*p*‐Value	Linear	Quadratic	Cubic	Model	*p*‐Value	Adj. *R* ^2^	*Y*_max_
Diet price (USD/kg)	1.93	1.70	1.48	1.25	1.03	0.80	—	—	—	—	—	—	—	—
ECR^1^ (USD/kg)	1.78 ± 0.022^a^	1.66 ± 0.051^a^	1.46 ± 0.046^b^	1.24 ± 0.047^c^	1.04 ± 0.022^d^	0.82 ± 0.012^e^	*p* < 0.0001	0.0001	0.1754	0.3919	*Y* = 0.0000005015*X* ^3^ − 0.00009666*X* ^2^ − 0.004942*X* + 1.2912	*p* < 0.0001	0.9718	—
EPI^2^ (USD/fish)	0.95 ± 0.006^a^	0.96 ± 0.027^a^	0.94 ± 0.013^a^	0.78 ± 0.019^b^	0.56 ± 0.018^c^	0.52 ± 0.018^c^	*p* < 0.0001	0.0001	0.0001	0.0003	*Y* = 0.000001431*X* ^3^ − 0.000263*X* ^2^ + 0.007631*X* + 0.9370	*p* < 0.0001	0.9490	16.81

*Note:* Values (means of triplicate ± SE) in the same row sharing the common superscript letter are not significantly different (*p*  > 0.05).

Abbreviations: Adj. *R* ^2^, adjusted *R* ^2^; Con, 60% fish meal (FM)‐based diet; DBM20, dietary 20% replacement of FM with DBM; DBM40, dietary 40% replacement of FM with DBM; DBM60, dietary 60% replacement of FM with DBM; DBM80, dietary 80% replacement of FM with DBM; DBM100, dietary 100% replacement of FM with DBM.

^1^Economic conversion ratio (ECR, USD/kg) = feed consumption (kg/fish)/weight gain (kg/fish) × diet price (USD/kg).

^2^Economic profit index (EPI, USD/fish) = [final weight (kg/fish) × selling price of fish (USD/kg)] – [feed consumption (kg/fish) × diet price (USD/kg)].

## 4. Discussion

The SGR (1.71%–3.04%/day) of juvenile olive flounder (initial weight of 6.3 g) supplied with the experimental diets in this study were comparable to those (2.8–3.4, 2.9–3.4, and 3.2%–3.6%/day) previously reported for the same species (initial weight of 6.3, 3.6, and 3.9 g, respectively) [32–34], indicating that olive flounder exhibited satisfactory growth performance. Superior WG and SGR of fish provided with the Con, DBM20, and DBM40 diets, with no statistically difference among these groups, compared to fish provided with the DBM60, DBM80, and DBM100 diets indicate that DBM can substitute up to 40% of FM in a 60% FM‐basal diet without statistically deteriorating WG and SGR in olive flounder. The *Y*_max_ values to maximize WG and SGR of fish were estimated to be 15.77% and 15.59% of the FM replacement with DBM in the diet, respectively. While dietary FM replacement up to 40% with DBM was sufficient to maintain similar growth performance compared with the Con diet, dietary optimal FM substitution levels with DBM for the maximum WG and SGR reached at 15.59%–15.77%.

Ha et al. [27] proved that 50% of FM in feeds could be replaced with CBM, one of PBM, without significantly lowering the growth performance of olive flounder when fish were supplied with a 65% FM‐based diet or diets with elevated FM replacement by CBM (10%–50%). However, up to 50% of FM in the diet could replace PBM without compromising the growth performance of European seabass when fish were supplied with a 50% FM‐based diet or diets in which FM was replaced by PBM at 25% increment levels (25%–100%) [35]. Unlike the present study, however, FM replacement with PBM in diets led to reduction in growth performance of gilthead seabream (*Sparus aurata*) when juvenile fish were supplied with a 58% FM‐based feed or feeds in which FM was replaced by PBM at 50% increment levels (50% and 100%) for 100 days [36]. Nevertheless, the same authors also demonstrated that up to 50% substitution of FM with PBM was feasible without impairing growth performance when fish were supplied with a 58% FM‐based feed or feeds in which 25% of FM was substituted with PBM supplemented with or without EAAs (lysine and methionine) or 50% of FM was replaced with PBM supplemented with EAAs, over a 110‐day feeding experiment.

Dietary requirements of arginine (2.04%–2.10% of the diet) [37], lysine (2.18%–2.39% of the diet) [38], threonine (1.03% of the diet) [39], and valine (1.77%–1.89% of the diet) [40] for olive flounder were all satisfied in this study. Nevertheless, inferior growth of fish fed the DBM60, DBM80, and DBM100 diets in comparison with those fed the Con diet indicates that dietary high (≥ 60%) FM substitution with DBM negatively affected growth performance. This may have resulted from decrease in other EAAs, except for arginine, lysine, threonine, and valine in the experimental diets as dietary FM replacements with DBM increased. Similarly, Siddik et al. [28] demonstrated that increasing dietary replacements of FM with PBM and bioprocessed PBM reduced EAAs availability, which in turn resulted in impaired growth in Asian seabass.

The substitutability of FM with PBM, including CBM and DBM in diets varies among various fish species, and relevant findings from previous studies are summarized in Table S1. Across different species, the reported levels of FM substitution with PBM range widely from 20% to 100%. The tolerable levels of FM substitution with PBM appears to depend largely on growth stage (body size). For instance, substitutability of FM with PBM increased with fish size, rising from 25% to 50% in black sea turbot (*Scophthalmus maeoticus*) with initial body weights of 18.0 and 30.2 g, respectively [41, 42]; from 60% to 100% in cobia (*Rachycentron canadum*) with initial body weight of 5.8 and 30.7 g, respectively [43, 44]; from 50% to 65% in gilthead seabream with initial body weight of 1.16 and 35.0 g, respectively [45, 46].

In addition, supplementation of deficient AAs in PBM in diets has been shown to enhance the substitutability of FM. For instance, 20% of FM could be replaced with PBM in a 58% FM‐based diet for greater amberjack (*Seriola dumerili*); however, supplementation with EAAs, including isoleucine, leucine, lysine, and methionine, increased the feasible FM replacement level to 40% [47]. Overall, these findings indicated that the substitutability of FM with PBM in fish feeds is strongly influenced by fish species, body size, and the supplementation of EAAs in PBM. Therefore, supplementation of specific EAAs may still be considered in future studies as a potential strategy to further improve the substitutability of FM with DBM.

Dietary n‐3 HUFA has been shown to improve growth and metabolic regulation in fish [48, 49]. However, marine teleost has a limited ability to convert C18 polyunsaturated FA (PUFA) into n‐3 HUFA, so it must be supplied through diets [50]. Dietary increased FM replacements with DBM led to decrease in ∑n‐3 HUFA, including EPA and DHA, which was well reflected from the FA profiles of the main ingredients (FM and DBM) in diets. An insufficiency of n‐3 long‐chain PUFA, including n‐3 HUFA, may account for growth reduction in fish [51]. Accordingly, the decrease in ∑n‐3 HUFA in diets with increasing substitution of FM by DBM might have negatively affected the growth of fish supplied with the DBM60, DBM80, and DBM100 diets. Similarly, complete substitution of FM with PBM reduced ∑n‐3 HUFA levels in the feeds and ultimately decreased WG and SGR in black sea bass (*Centropristis striata*) [52]. Likewise, substitution of 60%–80% of FM with combined animal proteins resulted in reduced ∑n‐3 HUFA levels in the feeds, which was accompanied by decline in final body weight and SGR of Japanese seabass (*Lateolabrax japonicus*) [53].

Olive flounder supplied with the Con, DBM20, and DBM40 diets exhibited discernible higher FC, with no statistical differences among these groups, than those supplied with the DBM60, DBM80, and DBM100 diets, indicating that up to 40% of FM could be substituted by DBM without adversely deteriorating FC. This pattern was closely associated with growth performance in this experiment. Moreover, the *Y*_max_ value required to induce the highest FC in olive flounder was estimated at 18.90% dietary FM replacement by DBM, which was consistent with the *Y*_max_ values estimated for WG and SGR. Similarly, increasing replacement levels of FM by PBM in the feeds reduced FC in gilthead seabream and ultimately decreased WG and SGR [36]. Likewise, complete substitution of FM by PBM lowered FC in European seabass, thereby impairing WG and SGR [35].

In addition, reduced FC observed in fish provided with the DBM60, DBM80, and DBM100 diets may be attributed to the high dietary ash content (15.5%−17.0%). Similarly, Prakash et al. [54] reported that elevated dietary ash levels reduced nutrient digestibility, thereby, impairing FC in rainbow trout (*Oncorhynchus mykiss*), and ultimately resulted in poorer growth. Likewise, dietary increased FM substitution with tuna by‐product meal was associated with increased dietary ash content, eventually lowering FC in red sea bream (*Pagrus major*) and ultimately leading to deteriorated growth performance [55]. FC was significantly reduced in Japanese seabass provide with the high‐calcium diets containing 31.0 g/Ca kg, relative to fish provided with the low‐calcium diets containing 2.9–10.2 g/Ca kg, accompanied by significantly lower WG and SGR [56].

Feed utilization is one of the most common indicators describing how feed is converted into body mass [8]. In this study, FE, PER, and PR were not statistically influenced by dietary treatments, demonstrating that growth performance of olive flounder was proportional to FC. Likewise, dietary substitution of FM with PBM resulted in no significant change in feed utilization in both gilthead seabream and hybrid grouper (*Epinephelus fuscoguttatus* ♀× *E. lanceolatus* ♂) [57, 58]. Similarly, dietary FM replacement with salmon by‐product meal or tuna by‐product meal, and meat and bone meal had no discernible impacts on FCR, PER, and FE of red sea bream and spotted rose snapper (*Lutjanus guttatus*), respectively [22, 59].

The CF of fish reflected the physical and biological conditions associated with variations in nutritional status, energy reserves, and physiological condition [60], and higher CF has been associated with greater market value and faster growth [61]. A significantly higher CF was found in olive flounder supplied with the Con diet than in those supplied with the DBM60, DBM80, and DBM100 diets, whereas it did not differ significantly from that of fish supplied with the DBM20 and DBM40 diets. Likewise, CF of hybrid grouper tended to decline with increasing FM replacement by PBM in an 80.45% FM‐based diet [58]. The VSI and HSI are widely used to evaluate fish nutritional condition, nutrient allocation, and physiological status [62]. The VSI and HSI of olive flounder were not significantly altered by dietary treatments in the present study. Previous studies have reported similar findings in rainbow trout and post‐smolt coho salmon (*O. kisutch*), in which dietary FM replacements with PBM did not affect either VSI or HSI [63, 64].

Plasma chemistry is widely used to evaluate metabolic and hepatic responses as well as health status of fish to dietary ingredient alternation [65, 66]. Plasma chemistry of fish was not statistically different among dietary treatments in the present study, indicating that complete substitution of FM with DBM in diets could be made without negative impact on plasma chemistry of fish. Similarly, plasma TP, TG, T‐CHO, glucose, and phospholipid did not differ significantly in milkfish (*Chanos chanos*) provide with a 20% FM‐based diet or diets substituting 40% and 60% of FM with PBM [67]. Dietary substitution of FM with PBM and bioprocessed PBM did not cause any significant differences in glucose, cholesterol, and TG of Nile tilapia (*Oreochromis niloticus*) [68]. Unlike this experiment, however, plasma TP, TG, T‐CHO, glucose, and low‐ and high‐density lipoprotein of hybrid grouper (*E. lanceolatus* ♂ × *E. fuscoguttatus* ♀) decreased with increased dietary substitution of FM with PBM [69].

The chemical composition of the whole‐body fish is widely used to evaluate the biological condition of fish, particularly in relation to growth and nutritional status [70, 71]. The experimental diets did not significantly affect the whole‐body chemical composition of olive flounder, indicating that dietary complete FM substitution with DBM could be made without causing any negative impact on the whole‐body chemical composition of fish. Similarly, replacing dietary FM with PBM did not affect the whole‐body chemical composition of gilthead seabream, and sobaity (*Sparidentex hasta*) [57, 72].

Several studies have reported the significant alterations in the AA profiles of whole‐body Asian seabass and gilthead seabream provided with diets in which various levels of FM were substituted with PBM [28, 45]. Isoleucine and tryptophan content of the whole‐body fish decreased quadratically and linearly, respectively, with increasing replacement of FM by DBM in the diet in this study. In contrast, glycine and proline content of the whole‐body fish exhibited a cubic increase. Similarly, all AA profiles of the whole‐body gilthead seabream were statistically altered, reflecting from the differences in the dietary AA profiles [45]. Likewise, dietary increased substitution of FM with PBM resulted in reduced histidine, leucine, lysine, methionine, serine, and tyrosine of the whole‐body hybrid grouper, which was well reflected from dietary FM replacements with PBM [69].

In the PCA analysis, one distinct separation (the Con diet) and two clusters (cluster 1 [DBM20, DBM40, and DBM60 diets], cluster 2 [DBM80 and DBM100 diets]) were identified. The whole‐body supplied fed the Con diet showed strong correlations with isoleucine, leucine, lysine, and tryptophan among EAAs, whereas cluster 2 was mainly associated with arginine and glycine. These results indicate that variation in whole‐body AA profiles was largely driven by differences in dietary AA profiles.

The ∑n‐3 HUFA of the whole‐body fish declined as dietary FM replacement with DBM increased, reflecting differences in FA profiles of the experimental diets. These results agree with previous reports, in which the whole‐body FA profiles of Asian seabass and black sea bass were closely reflected from dietary FA profiles when FM is replaced with PBM at different levels [28, 52]. Similarly, dietary FM replacements with PBM reduced ∑n‐3 PUFA, which was well reflected by a corresponding decrease in the whole‐body ∑n‐3 PUFA in Asian seabass [73]. Furthermore, increasing FM substitution with salmon by‐product meal decreased dietary EPA and DHA levels, leading to reduced EPA and DHA in the whole‐body of red sea bream [22].

In the PCA analysis, one distinct separation (the Con diet) and two clusters (cluster 1 [DBM20, DBM40, and DBM60], cluster 2 [DBM80 and DBM100]) were identified. The whole‐body fish supplied with the Con diet showed strong correlations with ARA and ∑n‐3 HUFA, particularly DHA and EPA, whereas cluster 2 was mainly associated with ∑SFA and ∑MUFA. These results suggest that variation in whole‐body FA profiles was largely driven by differences in dietary FA profiles.

Economic feasibility should be evaluated to fully integrate alternative ingredients into sustainable aquaculture practices [74]. Accordingly, an economic analysis was conducted of this study to assess the substitutability of FM with DBM in feeds. As dietary FM replacements with DBM increased, their costs tended to decrease, leading to lower ECR. The EPI is considered a more comprehensive indicator for evaluating economic profitability, as it incorporates production yield, WG, FCR, feed cost, and fish sale price [19, 31]. In the present study, significantly higher EPI values were observed in fish provided with the Con, DBM20, and DBM40 diets, with no significant differences among these groups, compared with the DBM60, DBM80, and DBM100 diets. Therefore, up to 40% of FM substitution with DBM in a 60% FM‐based diet is expected to yield the highest economic return for olive flounder farmers.

## 5. Conclusion

Up to 40% of FM could be substituted with DBM in a 60% FM‐based diet without negative impacts on growth performance, FC, feed utilization, and biological indices of olive flounder, or EPI. Therefore, the DBM40 diet appears to be the most suitable feeding strategy for olive flounder farmers. However, further studies are needed to evaluate the feasibility of DBM‐substituted diets under commercial‐scale farming conditions and the substitutability of FM with combined DBM and other protein sources in olive flounder diets.

## Author Contributions

**Seong Woo Shin:** investigation, methodology, writing – original draft, data curation. **Sung Hwoan Cho:** methodology, project administration, conceptualization, writing – review & editing.

## Funding

This work was supported by the National Research Foundation of Korea (NRF) grant funded by the Korea Government (MSIT) (RS‐2026‐25477661).

## Ethics Statement

All experimental procedures were conducted in accordance with ethical regulations and were approved by the Institutional Animal Care and Use Committee (IACUC) of Korea Maritime and Ocean University (Busan Metropolitan City, Korea) (KMOU IACUC 2024‐06).

## Conflicts of Interest

The authors declare no conflicts of interest.

## Supporting Information

Additional supporting information can be found online in the Supporting Information section.

## Supporting information


**Supporting Information** Table S1: Substitutability of poultry by‐product meal (PBM), chicken by‐product meal (CBM), and duck by‐product meal (DBM) for fish meal (FM) in diets for various fish.

## Data Availability

Data will be made available upon request.
