# Supplementary material for: Impacts of Replacing Fish Meal With Duck By‐Product Meal in Diets on Growth Performance, Feed Utilization, and Economic Efficiency of Olive Flounder (Paralichthys olivaceus)
Source: Aquac Nutr. 2026 Jun 18;2026:3065283. doi: 10.1155/anu/3065283 (PMC13278370; doi:10.1155/anu/3065283)
Supplement: Supplementary file 1 — Supporting Information Table S1: Substitutability of poultry by‐product meal (PBM), chicken by‐product meal (CBM), and duck by‐product meal (DBM) for fish meal (FM) in diets for various fish. [file ANU-2026-3065283-s001.docx]

**Table S1**

Substitutability of poultry by-product meal (PBM), chicken by-product meal (CBM), and duck by-product meal (DBM) for fish meal (FM) in diets for various ﬁsh.

| Fish species | Replacers for FM | Recommended FM replacement  levels of diet (%) | FM inclusion levels in a FM-basal diet (%) | Fish size  (Grown from initial weight to final weight) | Criteria compared | Supplemented essential amino acids | References |
| --- | --- | --- | --- | --- | --- | --- | --- |
| Olive flounder  (*Paralichthys olivaceus*) | CBM | 50 | 65 | From 14.7 g to 55.4 g | WG, SGR, FE, PER, and PR |  | [1] |
|  | DBM | 40 | 60 | From 6.3 g to 27.5 g | WG, SGR, FE, PER, and PR |  | This study |
| Asian seabass  (*Lates calcarifer*) | PBM | 75 | 61 | From 3.8 g to 32.7 g | WG, SGR, and FCR |  | [2] |
|  | Bioprocessed PBM | 75 | 61 | From 3.8 g to 33.7 g | WG, SGR, and FCR |  |  |
| Black sea turbot  (*Scophthalmus maeoticus*) | PBM | 25 | 77 | From 18.0 g to 29.2 g | SGR, FCR, and PER |  | [3] |
|  |  | 50 | 77 | From 30.2 g to 70.2 g | SGR, FCR, and PER |  | [4] |
| Cobia  (*Rachycentron canadum*) | PBM | 60 | 50 | From 5.8 g to 140. 9 g | WG and SGR |  | [5] |
|  |  | 100 | 50 | From 30.7 g to 131.1 g | WG, SGR, FCR, and PER |  | [6] |
| Gilthead seabream  (*Sparus aurata*) | PBM | 50 | 73 | From 1.6 g to 12.1 g | WG, SGR, FE, and PER |  | [7] |
|  |  | 65 | 50 | From 35.0 g to 135.2 | WG, SGR, and FCR |  | [8] |
| European seabass  (*Dicentrarchus labrax*) | PBM | 50 | 50 | From 0.9 g to 17.0 g | WG, FCR, and PER |  | [9] |
|  |  | 60 | 47 | From 0.7 g to 6.9 g | WG, SGR, FCR, and PER |  | [10] |
| Rockfish  (*Sebastes schlegeli*) | CBM | 20 | 55 | From 2.4 g to 12.2 g | WG, SGR, FE, PER, and PR |  | [11] |
| Spotted rose snapper  (*Lutjanus guttatus*) | PBM | 50 | 53 | From 11.0 g to 32.7 g | WG, SGR, FCR, and PER |  | [12] |
| Sobaity  (*Sparidentex hasta*) | PBM | 55 | 61 | From 29.4 g to 59.3 g | WG, SGR, FCR, and PER |  | [13] |
| Totoaba  (*Totoaba macdonaldi*) | PBM | 67 | 65 | From 2.7 g to 53.2 g | WG |  | [14] |
| Coho salmon  (*Oncorhynchus kisutch*) | PBM | 20 | 40 | From 179.7 g to 540.2 g | SGR, FCR, and PER |  | [15] |
| Rainbow trout  (*Oncorhynchus mykiss*) | PBM | 20 | 47 | From 34.5 g to 171.2 g | WG and SGR |  | [16] |
|  |  | 33 | 45 | From 48.5 g to 122.5 g | SGR, FCR, and PER |  | [17] |
|  |  | 100 | 66 | From 1.47 g to 21.8 g | WG and FCR |  | [18] |
| Black sea bream  (*Acanthopagrus schlegeli*) | PBM | 40 | 40 | From 0.5 g to 5.4 g | WG, FE, and PER | Lysine and  methionine | [19] |
| Gilthead seabream  (*Sparus aurata*) | PBM | 50 | 58 | From 2.5 g to 32.9 g | WG, SGR, FCR, PER, and PR | Lysine and  methionine | [20] |
| Hybrid grouper  (*Epinephelus fuscoguttatus*♀ × *E. lanceolatus*♂) | PBM | 60 | 55 | From 80.2 g to 309.8 g | WG, SGR, FE, and PER | Lysine, methionine, and threonine | [21] |
| Japanese seabass  (*Lateolabrax japonicus*) | PBM | 80 | 40 | From 8.5 g to 56.4 g | WG and FCR | Methionine | [22] |
| Red porgy  (*Pagrus pagrus*) | PBM | 70 | 74 | From 4.2 g to 17.4 g | WG, SGR, FCR, and PER | Lysine and  methionine | [23] |
| Greater amberjack  (*Seriola dumerili*) | PBM | 20 | 58 | From 93.0 g to 159.3 g | WG, SGR, FE, and PER | None | [24] |
|  |  | 40 | 58 | From 93.0 g to 164.2 g | WG, FE, and PER | Isoleucine, leucine, lysine, methionine, and threonine |  |

WG: weight gain; SGR: specific growth rate; FCR: feed conversion ratio; FE: Feed efficiency; PER: protein efficiency ratio; PR: protein retention

**References**

1. M. S. Ha, K. W. Lee, J. Kim, A. Yun, H. S. Jeong, M. J. Lee, S. I. Baek, S. H. Cho, K. W. Kim, S. G. Lim, B. J. Lee, S. W. Hur, M. Son, and S. Lee, “Dietary Substitution Effect of Fish Meal with Chicken By-Product Meal on Growth, Feed Utilization, Body Composition, Haematology and Non-Specific Immune Responses of Olive Flounder (*Paralichthys olivaceus*),” *Aquaculture Nutrition* 27 (2021): 315–326.
2. M. A. B. Siddik, P. Chungu, R. Fotedar, and J. Howieson, “Bioprocessed Poultry By-Product Meals on Growth, Gut Health and Fatty Acid Synthesis of Juvenile Barramundi, *Lates calcarifer* (Bloch),” *PLoS One* 14 (2019): e0215025.
3. A. Türker, M. Yiğit, S. Ergün, B. Karaali, and A. Erteken, “Potential of Poultry By-Product Meal as a Substitute for Fishmeal in Diets for Black Sea Turbot *Scophthalmus maeoticus*: Growth and Nutrient Utilization in Winter,” *The Israeli Journal of Aquaculture–Bamidgeh* 57 (2005): 49–61.
4. M. Yiğit, M. Erdem, S. Koshio, S. Ergün, A. Türker, and B. Karaali, “Substituting Fish Meal with Poultry By-Product Meal in Diets for Black Sea Turbot *Psetta maeotica*,” *Aquaculture Nutrition* 12 (2006): 340–347.
5. Q. Zhou, J. Zhao, P. Li, H. Wang, and L. Wang, “Evaluation of Poultry By-Product Meal in Commercial Diets for Juvenile Cobia (*Rachycentron canadum*),” *Aquaculture* 322 (2011): 122–127.
6. I. Saadiah, A. M. Abol-Munafi, and C. M. Che Utama, “Replacement of Fishmeal in Cobia (*Rachycentron canadum*) Diets Using Poultry By-Product Meal,” *Aquaculture International* 19 (2011): 637–648.
7. I. Nengas, M. N. Alexis, and S. J. Davies, “High Inclusion Levels of Poultry Meals and Related By-Products in Diets for Gilthead Seabream *Sparus aurata* L.,” *Aquaculture* 179 (1999): 13–23.
8. N. Damir, M. Yildiz, S. Ofori-Mensah, and I. Aydin, “Effect of Poultry By-Product Meal as Replacement for Fish Meal in Diets of Gilthead Seabream (*Sparus aurata*) Juveniles,” *Turkish Journal of Fisheries and Aquatic Sciences* 25 (2025): TRJFAS26756.
9. Y. Marzouk, M. M. Gaber, I. Ahmad, I. Ahmed, M. F. El Basuini, M. A. Zaki, A. E. M. Nour, E. M. H. Labib, and H. S. Khalil, “Impacts of Poultry By-Product Meal Substituting Fishmeal on Growth Efficiency, Body Composition, Liver, and Intestine Morphology of European Sea Bass *Dicentrarchus labrax*,” *Food Chemistry: X* 23 (2024): 101569.
10. T. M. Srour, M. A. Essa, M. M. Abdel-Rahim, and M. A. Mansour, “Replacement of Fish Meal with Poultry By-Product Meal (PBM) and Its Effects on the Survival, Growth, Feed Utilization, and Microbial Load of European Seabass, *Dicentrarchus labrax* Fry,” *Global Advanced Research Journal of Agricultural Science* 5 (2016): 293–301.
11. R. Li and S. H. Cho, “Fish Meal Replacement by Chicken By-Product Meal in Diet: Impacts on Growth and Feed Availability of Juvenile Rockfish (*Sebastes schlegeli*), and Economical Analysis,” *Animals* 15 (2025): 80.
12. C. Hernández, L. Osuna-Osuna, A. Benitez-Hernandez, J. Sanchez-Gutierrez, B. González-Rodríguez, and P. Dominguez-Jimenez, “Replacement of Fish Meal with Poultry By-Product Meal Feed Grade in Diet for Juvenile of Spotted Rose Snapper *Lutjanus guttatus*,” *Latin American Journal of Aquatic Research* 42 (2014): 111–120.
13. F. Hekmatpour, P. Kochanian, J. G. Marammazi, M. Zakeri, and S. Mousavi, “Inclusion of Poultry By-Product Meal in the Diet of *Sparidentex hasta*: Effects on Production Performance, Digestibility and Nutrient Retention,” *Animal Feed Science and Technology* 241 (2018): 173–183.
14. D. B. Zapata, J. P. Lazo, S. Z. Herzka, and M. T. Viana, “The Effect of Substituting Fishmeal with Poultry By-Product Meal in Diets for *Totoaba macdonaldi* Juveniles,” *Aquaculture Research* 47 (2016): 1778–1789.
15. H. Yu, M. Li, L. Yu, X. Ma, S. Wang, Z. Yuan, and L. Li, “Partial Replacement of Fishmeal with Poultry By-Product Meal in Diets for Coho Salmon (*Oncorhynchus kisutch*) Post-Smolts,” *Animals* 13 (2023): 2789.
16. H. Sevgİlİ and M. M. Ertürk, “Effects of Replacement of Fish Meal with Poultry By-Product Meal on Growth Performance in Practical Diets for Rainbow Trout, *Oncorhynchus mykiss*,” *Akdeniz Üniversitesi Ziraat Fakültesi Dergisi* 17 (2004): 161–167.
17. A. Keramat Amirkolaie, M. Shahsavari, and M. Hedayatyfard, “Full Replacement of Fishmeal by Poultry By-Product Meal in Rainbow Trout, *Oncorhynchus mykiss* (Walbaum, 1972) Diet,” *Iranian Journal of Fisheries Sciences* 13 (2014): 1069–1081.
18. G. Parés-Sierra, E. Durazo, M. A. Ponce, D. Badillo, G. Correa-Reyes, and M. T. Viana, “Partial to Total Replacement of Fishmeal by Poultry By-Product Meal in Diets for Juvenile Rainbow Trout (*Oncorhynchus mykiss*) and Their Effect on Fatty Acids from Muscle Tissue and the Time Required to Retrieve the Effect,” *Aquaculture Research* 45 (2014): 1459–1469.
19. M. Irm, S. Taj, M. Jin, J. Luo, H. J. T. Andriamialinirina, and Q. Zhou, “Effects of Replacement of Fish Meal by Poultry By-Product Meal on Growth Performance and Gene Expression Involved in Protein Metabolism for Juvenile Black Sea Bream (*Acanthoparus schlegelii*),” *Aquaculture* 528 (2020): 735544.
20. I. T. Karapanagiotidis, P. Psofakis, E. Mente, E. Malandrakis, and E. Golomazou, “Effect of Fishmeal Replacement by Poultry By-Product Meal on Growth Performance, Proximate Composition, Digestive Enzyme Activity, Haematological Parameters and Gene Expression of Gilthead Seabream (*Sparus aurata*),” *Aquaculture Nutrition* 25 (2019): 3–14.
21. Z. Wang, X. Qian, S. Xie, and B. Yun, “Changes of Growth Performance and Plasma Biochemical Parameters of Hybrid Grouper (*Epinephelus lanceolatus* ♂ × *Epinephelus fuscoguttatus* ♀) in Response to Substitution of Dietary Fishmeal with Poultry By-Product Meal,” *Aquaculture Reports* 18 (2020): 100516.
22. Y. Wang, F. Wang, W. Ji, H. Han, and P. Li, “Optimizing Dietary Protein Sources for Japanese Sea Bass (*Lateolabrax japonicus*) with an Emphasis on Using Poultry By-Product Meal to Substitute Fish Meal,” *Aquaculture Research* 46 (2015): 874–883.
23. J. C. Hill, M. S. Alam, W. O. Watanabe, P. M. Carroll, P. J. Seaton, and A. J. Bourdelais, “Replacement of Menhaden Fish Meal by Poultry By-Product Meal in the Diet of Juvenile Red Porgy,” North American *Journal of Aquaculture* 81 (2019): 81–93.
24. F. Takakuwa, H. Fukada, H. Hosokawa, and T. Masumoto, “Availability of Poultry By-Product Meal as an Alternative Protein Source for Fish Meal in Diet for Greater Amberjack (*Seriola dumerili*),” *Aquaculture Science* 54 (2006): 473–480.
